# Devant une tumeur mandibulaire: penser à une métastase pulmonaire

**DOI:** 10.11604/pamj.2015.22.262.8265

**Published:** 2015-11-19

**Authors:** Ines Kéchaou, Imène Boukhris

**Affiliations:** 1 Service de Médecine Interne B, Hôpital Charles Nicolle, Tunis, Tunisie

**Keywords:** Cancer, poumon, mandibule, Cancer, lung, mandible

## Image en medicine

L'articulation temporo-maxillaire est une localisation métastatique rare. Le primitif est classiquement prostatique ou mammaire. La localisation pulmonaire étant rare. Le type histologique le plus fréquemment est l'adénocarcinome. Le carcinome pulmonaire à petites cellules avec métastase mandibulaire est exceptionnel. Nous rapportons l'observation d'un patient âgé de 62 ans, tabagique à 40 paquet/année, admis pour une altération récente de l’état général évoluant depuis 1 mois. A l'examen, il avait une tuméfaction de consistance dure temporo-mandibulaire droite. L'examen respiratoire trouvait des râles ronflants aux 2 champs pulmonaires et un hippocratisme digital. Au bilan biologique, il y avait une VS à 55mm, une fibrinémie à 6,94g/l, une CRP à 25mg/l et une cholestase hépatique anictérique. L’échographie des parties molles de la tuméfaction faciale avait montré une masse tissulaire avec ostéolyse du Ramus mandibulaire. Le scanner du massif facial avait trouvé une lésion tissulaire lytique, d'allure secondaire, hypervasculaire, mal limitée, hétérogène, centrée sur la branche montante droite de la mandibule, étendue au condyle, mesurant 32x37 mm et envahissant les muscles masséters et ptérygoïdien. Le scanner thoraco-abdominal avait montré 2 masses tissulaires spiculées du lobe pulmonaire supérieur droit d'allure primitive, mesurant 30x18 mm. Il y'avait 2 lésions hypodenses hétérogènes intéressant les segments IV et VII du foie respectivement de 24 et 54 mm de grand axe. La biopsie pulmonaire scanno-guidée avait conclu à un carcinome neuroendocrine, à petites cellules, primitif pulmonaire. Le diagnostic de cancer pulmonaire avec métastases hépatiques et mandibulaire a été retenu. Le patient a été adressé en carcinologie médicale pour radio et chimiothérapie.

**Figure 1:**
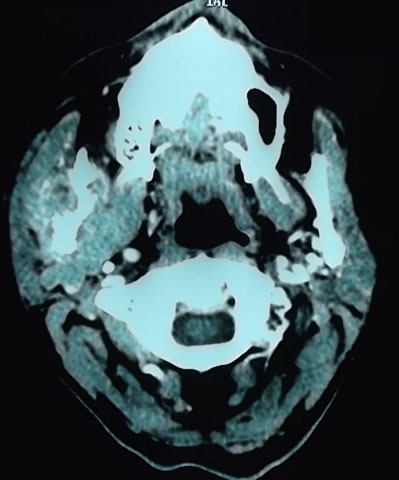
Lésion tissulaire lytique d'allure secondaire, hypervasculaire, mal limitée, de rehaussement hétérogène, centrée sur la branche montante droite de la mandibule et envahissant les muscles masséters et ptérygoïdien

